# The association between clinical integration of care and transfer of veterans with acute coronary syndromes from primary care VHA hospitals

**DOI:** 10.1186/1472-6963-5-2

**Published:** 2005-01-13

**Authors:** Anne E Sales, Sandra L Pineros, David J Magid, Nathan R Every, Nancy D Sharp, John S Rumsfeld

**Affiliations:** 1 Health Services Research and Development, VA Puget Sound Health Care System, Seattle WA, USA; 2 Department of Health Services, University of Washington, Seattle, WA, USA; 3 Denver VA Medical Center, Denver, CO, USA; 4 Colorado Permanente Clinical Research Unit, Denver, CO, USA; 5 Cardiology Service, VA Puget Sound Health Care System, Seattle, WA, USA; 6 Department of Medicine, University of Washington, Seattle, WA, USA

## Abstract

**Background:**

Few studies report on the effect of organizational factors facilitating transfer between primary and tertiary care hospitals either within an integrated health care system or outside it. In this paper, we report on the relationship between degree of clinical integration of cardiology services and transfer rates of acute coronary syndrome (ACS) patients from primary to tertiary hospitals within and outside the Veterans Health Administration (VHA) system.

**Methods:**

Prospective cohort study. Transfer rates were obtained for all patients with ACS diagnoses admitted to 12 primary VHA hospitals between 1998 and 1999. Binary variables measuring clinical integration were constructed for each primary VHA hospital reflecting: presence of on-site VHA cardiologist; referral coordinator at the associated tertiary VHA hospital; and/or referral coordinator at the primary VHA hospital. We assessed the association between the integration variables and overall transfer from primary to tertiary hospitals, using random effects logistic regression, controlling for clustering at two levels and adjusting for patient characteristics.

**Results:**

Three of twelve hospitals had a VHA cardiologist on site, six had a referral coordinator at the tertiary VHA hospital, and four had a referral coordinator at the primary hospital. Presence of a VHA staff cardiologist on site and a referral coordinator at the tertiary VHA hospital decreased the likelihood of any transfer (OR 0.45, 95% CI 0.27–0.77, and 0.46, p = 0.002, CI 0.27–0.78). Conversely, having a referral coordinator at the primary VHA hospital increased the likelihood of transfer (OR 6.28, CI 2.92–13.48).

**Conclusions:**

Elements of clinical integration are associated with transfer, an important process in the care of ACS patients. In promoting optimal patient care, clinical integration factors should be considered in addition to patient characteristics.

## Background

Coronary artery disease is the leading cause of death among Americans [1]. Hospitalization for acute coronary syndromes (ACS), which includes both acute myocardial infarction (AMI) and unstable angina, is common and costly. Many patients admitted with ACS to primary hospitals (i.e. those without on-site cardiology subspecialty services, including cardiac catheterization facilities) are transferred to tertiary hospitals for cardiac catheterization and consideration of coronary revascularization.

The coordination and integration between primary and tertiary hospitals has important implications for integrated health care delivery systems. The Veterans Health Administration (VHA) is one of the largest vertically integrated health care delivery systems in the United States [2]. The VHA is organized in 21 regional networks. Regionalization has been adopted by many integrated health care delivery systems, both to improve quality and to increase efficiency [3-6]. In most VHA regions, a single tertiary hospital is associated with one or more primary hospitals. A particular challenge in the VHA is providing access to sub-specialty cardiology services for patients hospitalized with acute coronary syndromes because primary hospitals are often geographically distant from tertiary hospitals [5].

Treatment guidelines for acute coronary syndromes [7-10] suggest that some diagnostic tests and therapies can be performed at most primary VHA hospitals, while others, such as cardiac catheterization and coronary revascularization, require transfer to a tertiary hospital. Well-functioning transfer processes are critical to making a policy of regionalization work. In addition, there are strong financial and organizational incentives to provide care within an integrated health care system like VHA rather than referring to non-VHA hospitals, even when this requires transfer to distant tertiary hospitals [11]. In the VHA, transfers within the system represent cost savings, while transfers out, by and large, represent cost increases. In addition to cost issues, there are also coordination of care concerns that are addressed through within-system transfer, particularly in a system with a common electronic medical record. However, the constraint on within-system transfer is that patients requiring urgent or emergent transfer to receive definitive care should be transferred to the nearest facility with capacity to provide care, even if this requires a transfer out of the system. Issues related to cost differences due to transfer within and outside integrated health care systems are most applicable in the United States, where the multiplicity of payers is a major financial concern; in other countries with integrated national health care, or single payer, systems, these issues are less relevant, although issues of care coordination may still be important.

The objective of this study was to evaluate the association between structural components of clinical integration and patient transfer rates from VHA primary hospitals to tertiary hospitals, both within and outside the VHA system for patients with ACS. We hypothesized that primary VHA hospitals with structural components of clinical integration present would have a higher rate of within-system transfer of ACS patients than primary VHA hospitals lacking these components.

## Methods

The VHA Access to Cardiology study was a prospective cohort study of 2,733 patients with a primary discharge diagnosis of either acute myocardial infarction (ICD9-CM 410.xx) or unstable angina (ICD9-CM 411.xx) discharged over a one year period (March 1, 1998 through February 28, 1999) from 24 VHA hospitals in five regions, including Minnesota and the Dakotas, the Southwest, the Rocky Mountains, the Pacific Northwest, and Southern California. Patient demographics, clinical characteristics, and specific processes of care including hospital transfer were obtained as part of the Access to Cardiology Study.

All patients admitted to one of the 12 primary VHA hospitals in the study were eligible for this analysis (n = 862 out of the 2,733 in the larger Access to Cardiology study). The remaining 12 VHA Medical Centers were tertiary hospitals with cardiology services and cardiac catheterization laboratories on site. These were not the focus of the analysis reported in this paper. We excluded 107 patients because they were initially admitted to a private hospital and transferred into a primary VHA hospital. In addition, we excluded 3 patients who were transferred from one primary VHA hospital to another. Finally, 27 patients had missing data in the variable indicating prior history of congestive heart failure, which was included in the final analysis. As a result, a total of 725 patients from 12 primary VHA hospitals were included in these analyses. The study protocol was approved by the Human Subjects Committee at the University of Washington, and by Institutional Review Boards and Research and Development Committees at each participating VHA hospital.

### Transfer rates

Patient transfer from a primary VHA hospital to a tertiary hospital (either VHA or private) was the primary outcome for this study. Secondary outcomes included both transfer from a primary VHA hospital to a tertiary VHA hospital, and transfer from a primary VHA hospital to a private (non-VHA) tertiary hospital.

Transfers to a tertiary VHA hospital were considered transfers within the system, while transfers to a private hospital were considered transfers outside the system. Transfer data were available for all 725 patients in the study cohort. We constructed two binary variables for the analyses: transfer to any tertiary care hospital (yes/no), and transfer to a tertiary VHA hospital versus transfer to a private (non-VHA) hospital.

### Clinical integration

The key independent variable for this study was clinical integration of cardiac services. We defined clinical integration [12,13] as the extent to which patient care services, in this case cardiology consultation services, are coordinated across the units and hospitals in the VHA providing care to cardiology patients. We measured clinical integration of cardiac services using three binary variables to indicate the presence or absence of these structural elements of clinical integration: a) a VHA staff cardiologist on-site at least episodically at the primary VHA hospital (either through a full or part time VHA staff cardiologist on site, or through periodic visits by a VHA staff cardiologist from the affiliated tertiary VHA hospital); b) a referral coordinator at the tertiary referral VHA hospital; and c) a referral coordinator at the primary VHA hospital. Referral coordinators at primary VHA hospitals are generalists, in that they facilitate referrals, transfers, and sometimes consultations for patients with many different kinds of diseases or health problems. In contrast, at tertiary VHA hospitals, referral coordinators are often associated with particularly sub-specialties, and work closely with these specialty services to provide assistance to referring hospitals and providers in determining whether transfer, referral, or consultation is advisable, and expediting the processes. These were all hospital level variables. We combined the two groups, VHA staff cardiologist on site and periodic visits by a VHA staff cardiologist, for two reasons. First, only one of the 12 primary hospitals in the sample had an on site VHA cardiologist, and the sample size in that group was too small to analyze independently. Second, in our interviews with Chiefs of Cardiology at the tertiary VHA hospitals, there was unanimity in their beliefs that either type of VHA cardiologist being available in a primary hospital produced more appropriate referrals, and improved interactions between providers at the primary hospital and the VHA tertiary cardiology service.

The data used to construct these measures came from on-site interviews conducted with Chiefs of Cardiology at each of the tertiary VHA hospitals associated with the primary VHA hospitals included in this study. During on-site interviews, Chiefs of Cardiology were asked to describe all of the primary VHA hospitals that refer ACS patients to them on a regular basis, and to identify the presence or absence of each of the structural elements of clinical integration. Interviews followed a structured protocol, ensuring uniform data collection. In all cases, the Chiefs of Cardiology were able to provide detailed information about the services available at both the tertiary and primary VHA hospitals. We also asked the Chief of Cardiology about the degree of competitiveness for cardiac services in the local markets for each of the primary VHA hospitals. This was an ordinal variable, with three levels: non-competitive; moderately competitive; or highly competitive market. In all cases, the Chief of Cardiology was able to answer the questions about market competition in the primary hospital market without difficulty, indicating considerable awareness of market conditions and the impact these had on their referral base.

In addition, we constructed two separate variables to control for patient distance from the primary VHA hospital to which they were initially admitted, and to control for the distance between primary and tertiary VHA hospitals. The patient distance variable was measured as the distance from the patient's home zip code centroid to the primary VHA hospital. The distance between the primary and tertiary referral VHA hospitals was measured in miles using VHA national databases. We tested different specifications of the distance variables, concluding that it was best to enter the distance between primary and tertiary VHA hospital as a continuous variable, whereas it made no difference in the results of the estimation what form we used for patient distance to primary VHA hospital. In the final analyses, it was dichotomized at greater than or equal to 100 miles – approximately two hours driving time. The patient distance variable is measured at the patient level, while the hospital distance variable is measured at the hospital level.

We included several measures of patient clinical characteristics, including age 65 or over; prior history of chronic obstructive pulmonary disease, bleeding disorder (such as hemophilia or anticoagulation therapy), smoking, prior percutaneous coronary intervention (PCI), or chronic heart failure; having a "Do Not Resuscitate" order, and several measures of seriousness or urgency of condition during the index admission in the primary VHA hospital: ST segment elevation on electrocardiogram or elevated cardiac enzymes at presentation; and a composite variable indicating the presence of a serious event during admission. Presence of a serious event during admission was a binary variable taking the value "1" if at least one of the following conditions was present: angina persisting more than 24 hours after admission; hypotensive episode; heart failure during admission; cardiac arrest; or positive stress test during admission. All of these variables were abstracted from the medical record.

### Analyses

We explored the bivariate association between clinical integration variables, distance variables, patient characteristic variables, and patient transfer using one-way analysis of variance with Scheffe correction for multiple comparisons.

To construct the most parsimonious models using the full set of candidate independent variables (clinical integration variables and patient characteristics), we used backward stepwise logistic regression, beginning with all available patient clinical characteristics that have been shown to be significant in predicting mortality outcomes for ACS patients in prior studies. We eliminated variables from the model if the p-value for the variable was greater than 0.1. A number of the candidate variables, including many of the history and co-morbidity variables, were found to be insignificant, and we created a summary variable described above which included many of the highly significant variables from the index hospital admission (details available from authors). C-statistics for each of the final models ranged from 0.77 to 0.85. We used Stata SE version 8.2 for all analyses.

We then investigated the relationship between clinical integration of cardiac services and transfer rates using random effects logistic regression [14], correcting for cluster sampling by hospital and region and controlling for distance and patient characteristics that reflect cardiac disease severity and therefore may affect the likelihood of transfer. Two models were estimated, one for transfer to any tertiary care hospital, and the second to estimate the conditional probability that the patient was transferred to a VHA tertiary hospital versus transfer to a non-VHA tertiary hospital, given that they were transferred. Random effects logistic regression allowed us to control for the effects of clustering on both the hospital and regional (Veterans Integrated Service Network, or VISN) level. The intra-class correlation of overall transfer with hospital and VISN jointly was 0.12 (p = 0.006), suggesting the need to control clustering at both levels.

## Results

Among the 12 primary VHA hospitals included in the sample, the mean rate of transfer was 42% (319 of 725). Mean rate of transfer to a tertiary VHA hospital was 31% (237 of 725), and to a private hospital was 11% (82 of 725). Most patients were transferred in order to receive cardiac catheterization or coronary revascularization. In addition, 37% of patients were treated in primary VHA hospitals that were over 250 miles from their tertiary referral VHA hospital, and 18% of patients lived over 100 miles from the primary VHA hospital to which they were admitted.

**Figure 1:**
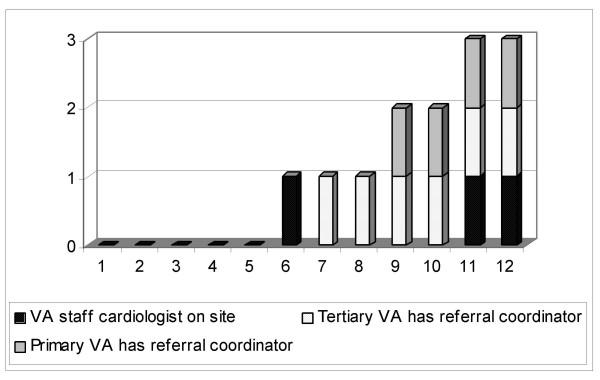
Distribution of integration components across the 12 primary VHA hospitals

Three of the 12 primary care VHA hospitals had a VHA cardiologist available at least episodically on site; six had a referral coordinator at the associated tertiary center; and four had a referral coordinator at the primary VHA hospital. The distribution of these components is shown in Figure 1. Five of the twelve hospitals had none of the three components of integration.

### Unadjusted associations

The bivariate associations between the patient characteristic variables, clinical integration variables, and type of transfer are shown in Table 1. All of the patient characteristics except history of chronic obstructive pulmonary disease were strongly and positively associated with transfer to a tertiary hospital. Distance between primary and tertiary VHA hospital was significantly different between the three groups, with overall transfer being associated with increased distance between the primary and tertiary VHA hospital. The degree of market competition was also significantly associated with transfer, principally to tertiary private hospitals. Each of the three individual components of integration were significantly associated with transfer from primary VHA.

**Table 1:** Patient and facility characteristics by transfer type

**Variable**	**Overall for study sample N = 755**	**Not transferred N = 436**	**Transferred to tertiary VHA hospital N = 237**	**Transferred to tertiary private hospital N = 82**	p-value*
**Patient age 65 and over**	58.0%	63.1%	52.3%	48.8%	0.005

**Prior medical history**					

**Chronic obstructive pulmonary disease**	37.1%	40.6%	31.9%	33.7%	0.067
**Bleeding disorder**	3.6%	2.1%	5.5%	6.2%	0.035
**Smoker**	31.6%	26.8%	41.8%	27.2%	<0.001
**Prior percutaneous coronary intervention**	15.2%	11.7%	21.5%	15.8%	0.003
**Chronic heart failure**	23.0%	28.9%	13.1%	18.8%	<0.001

**Course of index hospital admission**					

**ST segment elevation on EKG**	17.8%	12.8%	19.0%	39.0%	<0.001
**Cardiac enzymes abnormal on presentation**	52.5%	52.0%	46.4%	71.3%	<0.001
**Do not resuscitate during hospitalization**	5.3%	6.7%	2.1%	5.2%	0.039
In-hospital event**	47.3%	37.8%	62.9%	52.4%	<0.001

**Distance, market and integration variables**					

**Distance from patient home zip code centroid to hospital >100 miles**	18.1%	15.6%	21.1%	22.0%	0.128
**Distance from primary VHA to tertiary VHA hospital in miles**	281	270	285	326	0.045
**Degree of market competition (1 = not competitive; 3 = highly competitive)**	1.74	1.82	1.57	1.79	<0.001
**VHA cardiologist on site**	30.6%	29.8%	36.3%	19.5%	0.015
**Tertiary VHA hospital has referral coordinator**	54.7%	56.4%	60.8%	30.5%	<0.001
**Primary VHA hospital has referral coordinator**	33.0%	28.9%	43.9%	24.4%	<0.001

* p-value obtained from ANOVA testing difference between means for patients not transferred, transferred to VHA tertiary hospital, or transferred to non-VHA tertiary hospital for continuous variables, chi-square test of inference for categorical variables

** Presence of at least one of the following adverse events during admission: angina persisting more than 24 hours after admission; a hypotensive episode; an episode of heart failure; cardiac arrest; or positive stress test during admission

### Risk-adjusted association: transfer to any tertiary care hospital

Results of the random effects logistic regressions for transfer to any tertiary care hospital are shown in Table 2. Patient factors increasing the likelihood of transfer to a tertiary hospital included being a smoker; history of chronic heart failure; ST-segment elevation on presenting electrocardiogram; in-hospital events (presence of at least one of the following events during admission: angina persisting more than 24 hours after admission; a hypotensive episode; an episode of heart failure; cardiac arrest; or positive stress test during admission); and distance from patient home to hospital more than 100 miles.

**Table 2:** Results of random effects logistic regression of transfer to any tertiary care hospital

**Variable**	**Odds ratio**	**p-value**	**Lower limit 95% CI**	**Upper limit 95% CI**
**Patient age 65 and over**	0.69	0.06	0.48	1.01
**Chronic obstructive pulmonary disease**	0.48	<0.001	0.31	0.74
**Bleeding disorder**	0.68	0.04	0.47	0.98
**Smoker**	3.28	0.01	1.32	8.12
**Prior percutaneous coronary intervention**	1.30	0.18	0.89	1.91
**Chronic heart failure**	2.10	<0.001	1.33	3.32
**ST segment elevation on presenting electrocardiogram**	2.07	<0.001	1.32	3.26
**Cardiac enzymes abnormal on presentation**	0.92	0.65	0.64	1.31
**Do not resuscitate during hospitalization**	0.29	<0.001	0.12	0.65
In-hospital event*	3.14	<0.001	2.21	4.46
**Distance from patient home zip code centroid to hospital >100 miles**	1.71	0.02	1.08	2.70
**Distance from primary VHA to tertiary VHA hospital in miles**	0.998	0.03	0.997	0.999
**Degree of market competition (1 = not competitive; 3 = highly competitive)**	0.55	<0.001	0.41	0.73
**VHA cardiologist on site**	0.48	<0.001	0.29	0.79
**Tertiary VHA hospital has referral coordinator**	0.39	<0.001	0.23	0.69
**Primary VHA hospital has referral coordinator**	6.53	<0.001	3.29	12.98

* Presence of at least one of the following adverse events during admission: angina persisting more than 24 hours after admission; a hypotensive episode; an episode of heart failure; cardiac arrest; or positive stress test during admission

Patient factors that decreased the likelihood of transfer to any tertiary hospital included history of chronic obstructive pulmonary disease, or bleeding disorder; and having a do not resuscitate (DNR) order during the hospital admission. In addition, the further the distance between primary and tertiary VHA, the less likely patients were to be transferred at all, and the more competitive the market for cardiac care, the less likely that the patient was transferred to a tertiary care hospital.

All three components of integration were significantly associated with transfer to tertiary care, although in different directions. After adjustment for patient and other characteristics, the presence of a VHA staff cardiologist and having a referral coordinator at the tertiary VHA hospital decreased the likelihood of transfer to any tertiary care hospital. In contrast, the presence of a referral coordinator at the primary VHA hospital increased the probability of transfer to a tertiary hospital.

### Risk-adjusted association: transfer to tertiary VHA hospital vs. tertiary non-VHA hospital

The results of this analysis are shown in Table 3. Patient factors associated with transfer to VHA rather than private tertiary hospital included prior history of percutaneous coronary intervention, and history of chronic heart failure. Patient factors associated with transfer to private rather than VHA tertiary hospital included elevated ST-segment on presenting electrocardiogram, abnormal cardiac enzymes on presentation, and presence of a do not resuscitate order during the hospitalization.

**Table 3:** Results of conditional random effects logistic regression of transfer toVHA tertiary care compared to private tertiary care hospital

**Variable**	**Odds ratio**	**p-value**	**Lower limit 95% CI**	**Upper limit 95% CI**
**Patient age 65 and over**	1.42	0.29	0.75	2.71
**Chronic obstructive pulmonary disease**	0.56	0.15	0.25	1.23
**Bleeding disorder**	1.10	0.75	0.60	2.03
**Smoker**	1.14	0.84	0.34	3.77
**Prior percutaneous coronary intervention**	3.67	<0.001	1.91	7.04
**Chronic heart failure**	2.05	<0.001	1.43	2.95
**ST segment elevation on presenting electrocardiogram**	0.27	<0.001	0.14	0.51
**Cardiac enzymes abnormal on presentation**	0.30	0.02	0.11	0.81
**Do not resuscitate during hospitalization**	0.14	<0.001	0.04	0.54
In-hospital event*	1.47	0.31	0.70	3.08
**Distance from patient home zip code centroid to hospital >100 miles**	2.10	0.10	0.86	5.10
**Distance from primary VHA to tertiary VHA hospital in miles**	1.00	0.35	0.99	1.00
**Degree of market competition (1 = not competitive; 3 = highly competitive)**	0.19	0.06	0.03	1.05
**VHA cardiologist on site**	1.17	0.85	0.23	6.06
**Tertiary VHA hospital has referral coordinator**	20.62	<0.001	4.50	94.47
**Primary VHA hospital has referral coordinator**	1.38	0.69	0.27	6.99

* Presence of at least one of the following adverse events during admission: angina persisting more than 24 hours after admission; a hypotensive episode; an episode of heart failure; cardiac arrest; or positive stress test during admission

The degree of market competition was not significantly associated with transfer to VHA versus private tertiary hospital. Neither of the distance variables were associated with transfer either to VHA or non-VHA tertiary hospitals. Furthermore, only one of the individual integration variables entered separately were significantly associated with likelihood of transfer to tertiary VHA versus private hospital, and although the parameter estimate for the variable indicating presence of a referral coordinator at the tertiary hospital was large and significant, it was very imprecise (i.e. large standard error). This is probably due to the relatively small number of patients included in the estimation (N = 319) and uneven splits among hospitals, clustered by VISN.

## Discussion

The goal of this study was to investigate the association between measures of clinical integration of care and transfer of patients with acute coronary syndromes in the VHA. In particular, we evaluated whether structural components of clinical integration, such as the presence of referral coordinators and on-site cardiologists, were associated with patient transfer within and/or outside of the VHA healthcare system. In multivariate analysis, the presence of referral coordinators located at primary care VHA hospitals increased the overall likelihood of transfer of ACS patients. In contrast, having a VHA staff cardiologist available or a referral coordinator at a tertiary VHA hospital significantly decreased the likelihood of any transfer to a tertiary care hospital. Finally, we found that only one of the three integration components, presence of a referral coordinator at the tertiary VHA hospital, was significantly associated with transfer to a tertiary VHA hospital compared to a non-VHA tertiary hospital.

Our finding that referral coordinators at primary care hospitals increase the likelihood of transfer to tertiary care hospitals is consistent with prior studies demonstrating that referral coordinators increase the ease of referral and frequency of transfer [5,15-19]. Presence of a referral coordinator at the primary hospital means that a knowledgeable staff person, not a physician but usually a clinician such as a nurse, is available to coordinate and facilitate what can otherwise be a very cumbersome process of referral and transfer. This individual usually locates and communicates with tertiary care providers and facilitates paperwork and other processes required for patient transfer.

However, our finding that the presence of a referral coordinator at a tertiary VHA hospital was negatively associated with transfer appears contradictory. It is possible that referral coordinators at the tertiary centers may facilitate consultation, which may, at least for lower risk patients, appropriately reduce the need for transfer. However, it is of some concern that these referral coordinators may be serving in a gatekeeper role with regard to transfer decisions. Future research should focus on the role and decision-making associated with these referral coordinators. Of note, when transfer did occur, the presence of a referral coordinator at the tertiary VHA hospital was positively associated with transfer to VHA facilities rather than non-VHA facilities. This suggests that referral coordinators may function differently with different kinds of patients, decreasing overall transfer rates but facilitating within-system transfer when transfer occurred.

In general, we found that transfers to tertiary care were largely associated with patient characteristics appropriate to transfer: sicker and more urgent patients, except for those for whom more intensive care may not be indicated (e.g. DNR status), were significantly more likely to be transferred. In particular, patients with ST-segment elevation on their presenting electrocardiogram and abnormal cardiac enzymes were significantly more likely to be transferred, most likely for coronary revascularization. These patients are most likely to benefit from revascularization [5,9], and their higher probability of transfer suggests that appropriate triage and risk stratification took place in the primary VHA hospitals providing their care. In addition, we found that these patients were more likely to be transferred to non-VHA tertiary hospitals, presumably because these hospitals were closer to the primary VHA hospital than the affiliated tertiary VHA hospital, indicating appropriate out-of-system transfer for the most urgent patients who could benefit from rapid access to tertiary care. The finding that DNR status appears to be associated with transfer to private tertiary rather than VHA tertiary hospital may be due to small cell size, combined with other characteristics of the small number of patients with that status among those who were transferred at all (9 of 319).

Distance between the patient's home and primary VHA hospital was significantly associated with increased likelihood of subsequent transfer to a tertiary care hospital. This may indicate that patients who live further from the hospital take longer to present and are therefore sicker on arrival, leading to the requirement for higher levels of care. Also of interest, distance between primary and tertiary VHA hospitals was significantly associated with a decreased likelihood of transfer, indicating that in situations where primary and tertiary VHA hospitals are further apart, primary VHA hospitals may elect to keep more ACS patients rather than transfer them at all. Future research is needed on the appropriateness of transfer of ACS patients, as it is not clear that variation in transfer based on distance between hospitals represents appropriate variation in care.

The finding that cardiologist availability at the primary VHA hospitals was associated with less transfer to tertiary care hospitals may reflect that local or distant cardiology consultation was sufficient in some cases (e.g. lower risk patients) to avoid transfer. Similarly, the availability of a transfer coordinator at the tertiary VHA hospital may have provided an avenue for consultation and avoidance of transfer in some cases. Future studies are needed to define the mechanisms of association between reduced transfers and both on-site cardiology availability and tertiary hospital transfer coordinators.

The findings of this study, that referral coordination is associated with transfer from primary to tertiary hospitals, but may operate differently for different types of patients, and may have one mechanism of operation within a health care system and another outside that system, have potential application outside VHA. Previous studies [20] have found that patients' access to needed services, such as revascularization after acute myocardial infarction, has a significant effect on mortality outcomes. Services such as referral coordination, which increase the likelihood that a patient will be transferred, can reduce the negative impact of receiving initial care in a hospital without specialized tertiary services, such as cardiac catheterization. These findings are potentially relevant in all health care systems where hospitals have different levels of service. Even though they are based on a relatively small patient sample size, the implications of the findings – that referral coordinators at primary hospitals increase the probability of transfer, with the link to better outcomes at tertiary centers [21] with a full range of treatment options – should spark discussion in a health care system such as VHA about recommending use of referral coordinators in primary hospitals.

### Limitations

First, we were not able to conduct full-scale validation and reliability testing of the clinical integration measures, which would have required a larger sample of hospitals participating in the study to conduct split-sample validation. Second, we used structural, rather than process, elements of integration in this analysis. We focus on structural elements both because they are relatively easier to measure (present or not), and because in Donabedian's widely accepted model of quality in health care, structure precedes process and outcome [22,23]. Third, clinical integration is a complex multi-faceted construct which we captured in a relatively simplistic way. However, we wanted to see if measures that would be straightforward to implement in a health care system like the VHA, such as referral coordinators, had an impact on this key process of care. We measured other components of integration, including communication methods, provider satisfaction with communication methods, and overall perception of how well referral and consultation worked in providing care to ACS patients. Individually, these factors were not as strongly linked to the transfer process as the three structural components we present in this analysis.

Fourth, because transfer is closely related to patient outcomes, especially for ACS patients [21], careful modeling of the relationship between transfer and mortality and morbidity outcomes is essential. We plan to conduct future analyses on the relationships between patient characteristics, transfer, and mortality and morbidity outcomes. In addition, it is important to note that most veterans over the age of 65 are dually eligible for Medicare as well as VHA benefits, and previous analyses have shown that a majority of veterans with acute myocardial infarction, even among those who use VHA hospitals, receive care for AMI in private hospitals [24,25]. This study was designed only to assess transfer of veterans who went to primary VHA hospitals for their ACS care.

## Conclusions

We found that referral coordinators located at primary care VHA hospitals increase the overall likelihood of transfer of ACS patients. Referral coordinators at tertiary VHA hospitals and the presence of on-site cardiologists appeared to decrease the likelihood of transfer. Only one component of integration, presence of a referral coordinator at the tertiary hospital, was associated with within-system compared to out-of-system transfer. These findings have significant potential implications for the VHA. One of the goals of an integrated health care system is to maintain optimal coordination between its component parts [12]. This study demonstrates that simple structural components of care, such as a referral coordinator at either a primary or tertiary care hospital, can have an impact on a key process of care above and beyond patient characteristics.

## Competing interests

The author(s) declare that they have no competing interests.

## Authors' contributions

AES participated in the design and conduct of the study, conducted the analyses and wrote the manuscript. SLP participated in conducting the project, and assisted in writing the manuscript. DJM participated in writing the manuscript. NRE participated in the design and conduct of the study. NDS participated in writing the manuscript. JSR participated in the statistical analyses and co-wrote the manuscript. All authors read and approved the final manuscript.

## Pre-publication history

The pre-publication history for this paper can be accessed here:


